# Cytotoxicity of bendamustine, alone and in combination with novel agents, toward adult T-cell leukemia cells

**DOI:** 10.1371/journal.pone.0309533

**Published:** 2024-09-30

**Authors:** Naoki Osada, Jiro Kikuchi, Yosuke Okada, Sae Matsuoka, Kazuhiro Morishita, Hideki Nakasone, Yusuke Furukawa

**Affiliations:** 1 Division of Emerging Medicine for Integrated Therapeutics (EMIT), Center for Molecular Medicine, Jichi Medical University, Shimotsuke, Tochigi, Japan; 2 Division of Hematology, Jichi Medical University Saitama Medical Center, Saitama, Japan; 3 HTLV-1/ATL Research, Education and Medical Facility, Faculty of Medicine, University of Miyazaki, Miyazaki, Japan; 4 Project for Advanced Medical Research and Development, Project Research Division, Frontier Science Research Center, University of Miyazaki, Miyazaki, Japan; 5 Center for Medical Education, Teikyo University of Science, Tokyo, Japan; University of Verona, ITALY

## Abstract

Adult T-cell leukemia/lymphoma (ATL) develops from the infection of T cells with human T lymphotropic virus type 1 (HTLV-1). There are an estimated 5–20 million HTLV-1 carriers worldwide and the patients are frequently observed in subtropical Africa, the Caribbean, Middle East, South America, and South West Japan. The prognosis of ATL remains dismal due to rapid acquired resistance to treatment with cytotoxic chemotherapeutic agents. In particular, the development of novel therapies for relapsed or refractory (R/R) ATL is an unmet need. Previous clinical trials revealed that bendamustine (BDM) was effective as the first-line treatment for indolent lymphoma and R/R cases of diffuse large B-cell lymphoma. Its major advantage is that it has few side effects such as hair loss and peripheral neuropathy, and does not impair the quality of life. However, its efficacy has not been verified for ATL in pre-clinical or clinical studies. In this study, we have shown the cytotoxicity of BDM alone and in combination with novel agents including the histone deacetylase (HDAC) inhibitor tucidinostat, the enhancer of zeste homolog 1/2 (EZH1/2) dual inhibitor valemetostat, and the Bcl2 family inhibitor ABT-737. The combined *in vitro* effects of BDM and tucidinostat were reproduced in a murine model without any obvious hematological toxicity. Our present results suggest that the combination of tucidinostat and BDM could additively prolong the survival of patients with R/R progressive ATL. The efficacy and safety of this combination are thus worthy of investigation in clinical settings.

## Introduction

Adult T-cell leukemia/lymphoma (ATL) is a hematological malignancy that develops from the infection of T cells with human T lymphotropic virus type 1 (HTLV-1). There are an estimated 5–20 million HTLV-1 carriers worldwide, especially in subtropical Africa, the Caribbean, Middle East, South America, and South West Japan [1]. ATL is more common in individuals who acquired HTLV-1 through lactation in childhood, although these individuals generally go through a 5–60 year asymptomatic carrier period before developing ATL [2].

ATL can be classified as acute, lymphoma, chronic, and smoldering types, with a median survival of 6.2, 10.2, 24.3 months, and not reached, respectively, for patients diagnosed between 2000 and 2009 [3]. ATL rapidly acquires resistance to treatment with cytotoxic chemotherapeutic agents and the prognosis remains dismal. HTLV-1-infection increases the genomic instability of T cells [4], suggesting that the accumulation of genomic and epigenomic changes might confer rapidly acquired drug resistance to ATL. Allogeneic hematopoietic stem cell transplantation may lead to long-term remission, but its indications are limited due to high non-relapse mortality and the age of the patients [3]. In particular, relapsed or refractory (R/R) ATL is an unmet need for the development of novel therapies to improve patient outcomes.

Bendamustine (BDM) is a multifunctional alkylating agent that contains three elements: a 2-chloroethylamine alkylating group, a benzimidazole ring, and a butyric acid side chain. The anti-tumor activity of BDM is reported to be different from that of other alkylating agents because of its unique benzimidazole ring [5]. Previous studies have shown the activation of DNA damage response and subsequent apoptosis, inhibition of mitotic checkpoints, and induction of mitotic catastrophe as the mechanisms of action of BDM [6]. BDM is rapidly incorporated into target cells through nucleoside transporters, probably because of its purine-like structure, thereby inducing DNA damage significantly faster than other agents [7].

BDM is one of the first-line agents for indolent lymphoma and has also been effective in R/R cases of diffuse large B-cell lymphoma (DLBCL). Compared with cyclophosphamide, doxorubicin, vincristine, and prednisolone also known as the CHOP regimen, the conventional standard of care for non-Hodgkin’s lymphoma, it is as effective or more effective as a single agent. In addition, its major advantage is that its few side effects are generally mild, such as hair loss and peripheral neuropathy, and do not impair the quality of life [8]. On the other hand, BDM increased the risk of opportunistic infections in association with CD4-positive lymphopenia due to specific cytotoxicity of BDM toward CD4-positive cells [9]. Then, this prompted us to speculate that BDM could be an effective agent for CD4-positive cell cancers including ATL. However, its efficacy has not been verified for ATL in pre-clinical or clinical studies.

In this study, we aimed to determine the cytotoxicity of BDM alone and in combination with novel agents including the histone deacetylase (HDAC) inhibitor tucidinostat [10], the enhancer of zeste homolog 1/2 (EZH1/2) dual inhibitor valemetostat [11], and the Bcl2 family inhibitor ABT-737 [12].

## Materials and methods

### Drugs

We purchased BDM, tucidinostat, valemetostat, and ABT-737 from Selleck Chemicals (Houston, TX, USA). Drugs were dissolved in dimethyl sulfoxide (DMSO) and used at a final dilution of 1/1000 to keep the final concentrations of DMSO < 0.1% to prevent alterations of drug effects or cell growth.

### Cells and cell culture

We used four adult T cell leukemia/lymphoma (ATL) (Su9T01, S1T, SO4, and ATN-1) [13–15], two HTLV-1-infected T cell (MT2 and TLSu) [16, 17], four mantle cell lymphoma (MCL) (SMCH-16, HBL-2, Jeko-1, and Rec-1) [7], a diffuse large B-cell lymphoma (B104), three Burkitt lymphoma (BL) (Daudi, Ramos, and Raji) [7], and four multiple myeloma (MM) (MM.1S, KMS12-BM, RPMI8226, and U266) [7] cell lines for drug sensitivity screening. MT2 was a kind gift from Dr. Hidekatsu Iha (Oita University, Oita, Japan). SO4 was a kind gift from Dr. Yasuaki Yamada (Nagasaki University, Nagasaki, Japan). Su9T-01 and S1T were kind gifts from Dr. Naomichi Arima (Kagoshima University, Kagoshima, Japan). TLSu was a kind gift from Dr. Kazuhiro Morishita (Miyazaki University, Miyazaki, Japan). SMCH-16 was a kind gift from Dr. Shigehisa Mori (Saitama Medical University, Saitama, Japan). MM.1S was a kind gift from Dr. Hiroshi Yasui (The University of Tokyo, Tokyo, Japan; St. Marianna University School of Medicine, Kawasaki, Japan). ATN-1 was provided by the RIKEN BRC through the National Bio Resource Project of the MEXT, Japan. HBL-2, Jeko-1, Rec-1, B104, MM.1S, KMS12-BM, RPMI8226, and U266 were purchased from the Health Science Research Resources Bank (Osaka, Japan). These cell lines were maintained in RPMI1640 medium supplemented with 10% heat-inactivated fetal bovine serum (FBS) in a humidified atmosphere of 5% CO_2_ at 37°C. SO4 was maintained in RPMI1640 medium supplemented with 10% FBS and recombinant human IL-2 (100 IU/mL) [14]. The cell line authenticity and absence of mycoplasma contamination are routinely checked by DNA fingerprinting and PCR.

### Cell proliferation assays

Cell proliferation was estimated by the conversion of MTT (3-[4,5-dimethylthiazol-2-yl]-2,5- diphenyltetrazolium bromide) to formazan in viable mitochondria using a Cell Counting Kit (Wako Biochemicals, Osaka, Japan). In brief, cells were seeded in 96-well flat-bottomed microplates at a density of 1 × 104 cells per well and incubated at 37°C. After incubation, the absorbance of formazan was measured at a wavelength of 450 nm using a microplate reader (Bio-Rad Laboratories, Hercules, CA), and expressed as a percentage of the absorbance value of the corresponding control cells [18].

### Immunoblotting

Immunoblotting was carried out according to a standard method using the following antibodies: anti-cleaved caspase3 (#9661) (Cell Signaling Technology, Beverly, MA) and anti-GAPDH (sc-47724) (Santa Cruz Biotechnology, Santa Cruz, CA) [19].

### Drug combination studies

The combined effect of BDM was evaluated by the method of Chou and Talalay [20]. S1T, TLSu, MT2, and ATN-1 cells were treated with BDM in combination with tucidinostat, valemetostat, or ABT-737 for 72 h. Cell viability was determined by the MTT reduction assay to obtain dose-response curves for each combination. We generated isobolograms and calculated the combination index (CI) using CompuSyn software according to the manufacturer’s instructions (http://www.combosyn.com). Combination indexes (CI) <0.8 and 0.8–1.2 indicate synergism and additivity between two drugs, respectively [19].

### Xenograft murine ATL model

For *ex vivo* tracing of tumors, we established a luciferase-expressing subline of MT2 by transfecting firefly luciferase cDNA [21] and inoculated 5 × 10^5^ cells subcutaneously into the right thigh of male NOD/SCID mice (CLEA Japan, Shizuoka, Japan). Tumor-derived luciferase activity was measured *ex vivo* by the IVIS Imaging System with Living Image software (Xenogen, Alameda, CA) after D-luciferin injection. When tumors developed to over 5 × 10^5^ photons/s, we randomized the mice into four treatment groups (day 1). We excluded mice whose tumors did not develop to 5 x 10^5^ photons/s. Each group was treated with the vehicle alone (0.9% NaCl, orally, 5 times a week) (n = 5), tucidinostat alone (20 mg/kg, orally, 5 times a week) (n = 5), BDM alone (30 mg/kg, intraperitoneally, 3 times a week) (n = 3), or the combination of tucidinostat and BDM (n = 3) for 2 weeks. All animal studies were approved by the Institutional Animal Ethics Committee (approval ID: 24022–01, March 07, 2024) and performed in accordance with the Guide for the Care and Use of Laboratory Animals formulated by the National Academy of Sciences. (1) methods of sacrifice: All mice were sacrificed by CO2 or isoflurane anesthesia. (2) methods of anesthesia and/or analgesia: Mice were kept warm using heating pads and imaged and treated under anesthesia by 2% isoflurane anesthesi. (3) efforts to alleviate suffering: Throughout the experiments, mice were visually monitored and placed under warmed pads or warm heat lamps to prevent hypothermia. Caliper measurements of the longest perpendicular tumor diameters were performed to estimate tumor volume using the following formula: 4/3π x (width/2)2 x (length/2), which represents the three-dimensional volume of an ellipse. Mice were weighed and clinically observed throughout the experiment. Mice were defined as moribund and euthanized on the day if one of the following was observed: (1) persistent loss of body weight of >20%, (2) tumors that inhibit normal physiological function such as eating, drinking, and mobility, (3) open, ulcerated wounds that do not form scabs, (4) tumor volume >2000mm3, or (5) clinical observations of prostration, paralysis, seizures and hemorrhages.

### Immunofluorescence staining of tumor specimens

Subcutaneous tumors were resected from euthanized mice, weighed, and embedded in the Tissue Tek O.C.T. compound (Sakura Finetek, Tokyo, Japan) after formalin fixation. We prepared 4μm thick sections and subjected them to hematoxylin-eosin staining or immunohistochemical staining with rabbit anti-cleaved caspase-3 (#9661) (Cell Signaling Technology) antibodies as primary antibodies and Alexa Fluor 488 conjugated anti-rabbit IgG (Thermo Fisher Scientific) as secondary antibodies. Nuclei were counterstained with 4’,6-diamidino-2-phenylindole (DAPI) (#8961) (Cell Signaling Technology). The stained samples were examined under a BZ-X fluorescence microscope (Keyence, Osaka, Japan) [19].

### Statistics

We used KaleidaGraph software (Synergy Software) to perform one-way ANOVA with the Student-Newman-Keuls multiple comparison test and Student’s t-test to determine statistical significance. P-values less than 0.05 were considered significant.

## Results and discussion

**Fig 1:**
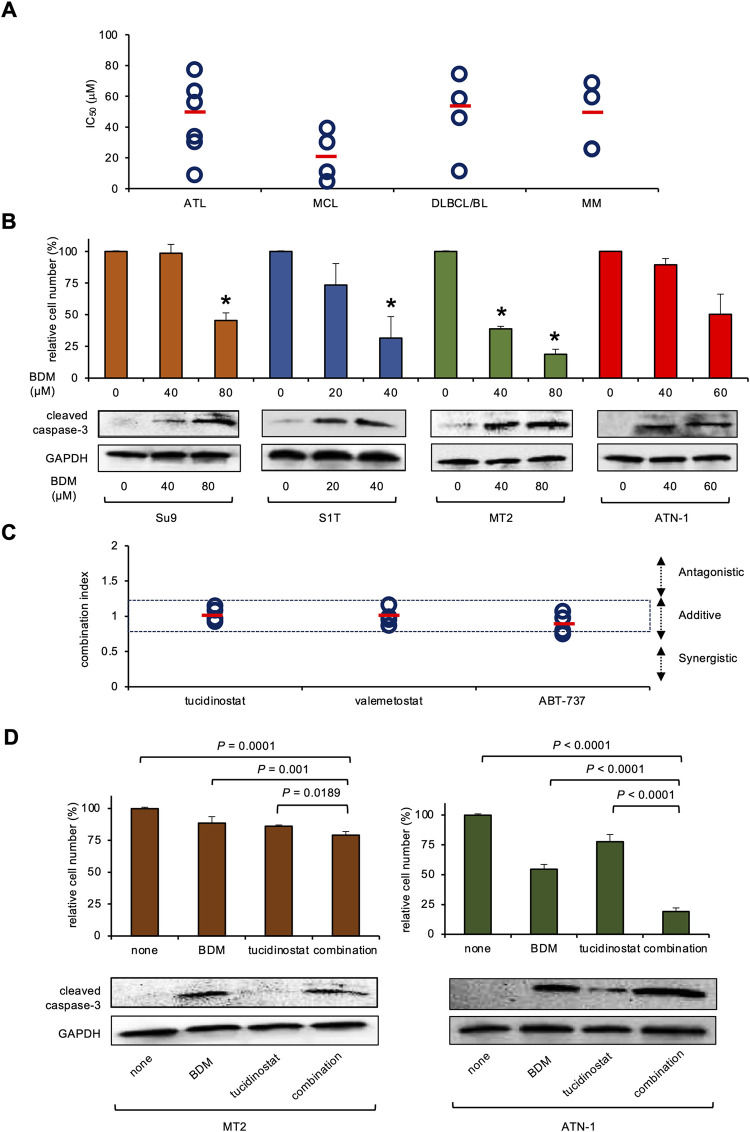
*In vitro* cytotoxicity toward ATL cells of bendamustine alone and in combination with novel agents. (A) We cultured the indicated cell lines with various concentrations of bendamustine (BDM) (0–100 μM) and cell viability was determined by the MTT (3-(4,5-dimethylthiazol-2-yl)-5-(3-carboxymethophenyl-2-(4-sulfophenyl)-2H-tetrazolium) reduction assay after 72 h to obtain dose-response curves. IC_50_ values are defined as the concentrations of drug that produce 50% inhibition of cell growth. (B) Upper panel; Su9, S1T, MT2 and ATN-1 cells were cultured with various concentrations of BDM for 72 h. Cell viability was determined by the MTT reduction assay and expressed as a percentage of the untreated control. The means ± S.D. (bars) of multiple independent experiments (n = 3) are shown. Asterisks indicate *P* < 0.05 vs untreated control. P values were determined by one-way analysis of variance (ANOVA) with a Student–Newman–Keuls multiple comparison test. Lower panel; Su9, S1T, MT2 and ATN-1 cells were cultured with various concentrations of BDM for 24 h. Whole cell lysates were simultaneously obtained and subjected to immunoblotting for cleaved caspase-3 and glyceraldehyde 3-phosphate dehydrogenase (GAPDH) proteins (loading control). (C) S1T, TLSu, MT2, and ATN-1 cells were treated with BDM in combination with tucidinostat, valemetostat, or ABT-737 for 72 h. Cell viability was determined by the MTT reduction assay to obtain dose-response curves for each combination. Combination index plots were generated by CompuSyn software. Combination indexes (CI) <0.8 and 0.8–1.2 indicate synergism and additivity between two drugs, respectively. Bars indicate the means of four ATL cell lines. (D) Upper panel: MT2 and ATN-1 cells were treated with vehicle alone (control), BDM (MT2: 40 μM, ATN-1: 80 μM), tucidinostat (MT2 and ATN-1: 150 nM), or a combination of both (combination) for 72 h. Cell viability was determined by the MTT reduction assay and expressed as a percentage of the untreated control. The means ± S.D. (bars) of multiple independent experiments (n = 3) are shown. P values were determined by one-way analysis of variance (ANOVA) with a Student–Newman–Keuls multiple comparison test. Lower panel: Whole cell lysates were simultaneously obtained and subjected to immunoblotting for cleaved caspase-3 and GAPDH (loading control) proteins.

We have shown the efficacy of BDM for mantle cell lymphoma (MCL), DLBCL, Burkitt lymphoma (BL), and multiple myeloma (MM) cell lines *in vitro* [7]. In the present study, we also determined the cytotoxicity of BDM for various hematological malignancies including ATL. The MTT assay revealed that the mean IC50 values of BDM were 44.9±25.0 μM, 21.1±16.2 μM, 47.5±26.8 μM, and 44.8±22.5 μM for ATL, MCL, DLBCL/BL, and MM cell lines, respectively (Fig 1A and S1A Fig). Although the IC_50_ values of DLBCL and MM were higher than those of MCL, the efficacy and safety of DLBCM and MM have been validated in clinical trials for DLBCL and MM [22, 23]. The IC_50_ values of BDM for ATL were similar to those of MM and DLBCL, suggesting that BDM might be clinically effective for ATL therapy. Immunoblot analyses revealed that BDM induced caspase-3 activation in ATL cell lines in a dose-dependent manner with kinetics similar to the induction of cellular cytotoxicity (Fig 1B). These results suggest that BDM induces caspase-induced apoptosis of ATL cells and hence, could be an effective agent for the treatment of ATL, as well as MCL, DLBCL, or MM.

Next, we determined the combined effect of BDM to enhance the cytotoxicity toward ATL. Recently, the histone deacetylase (HDAC) inhibitor tucidinostat and the enhancer of zeste homolog 1/2 (EZH1/2) dual inhibitor valemetostat have been approved for clinical use in R/R ATL in Japan [10, 11]. In addition, the Bcl2 family inhibitor ABT-737 exerted cytotoxicity toward ATL cells alone and in combination with conventional chemotherapeutic agents [12]. Thus, we analyzed the combined effects of BDM with these agents for the treatment of ATL using the isobologram method. Isobologram analyses revealed that the combination index of BDM and either tucidinostat, valemetostat, or ABT737 was approximately 1.0, suggesting an additive effect of the combination (Fig 1C and S1B Fig). Thus, the combination of BDM and these agents may be effective for the treatment of ATL. In particular, we determined the combined effect of BDM with tucidinostat for further study since we had shown that BDM additively enhanced HDAC inhibitor-mediated cytotoxicity in MCL [24].

**Fig 2:**
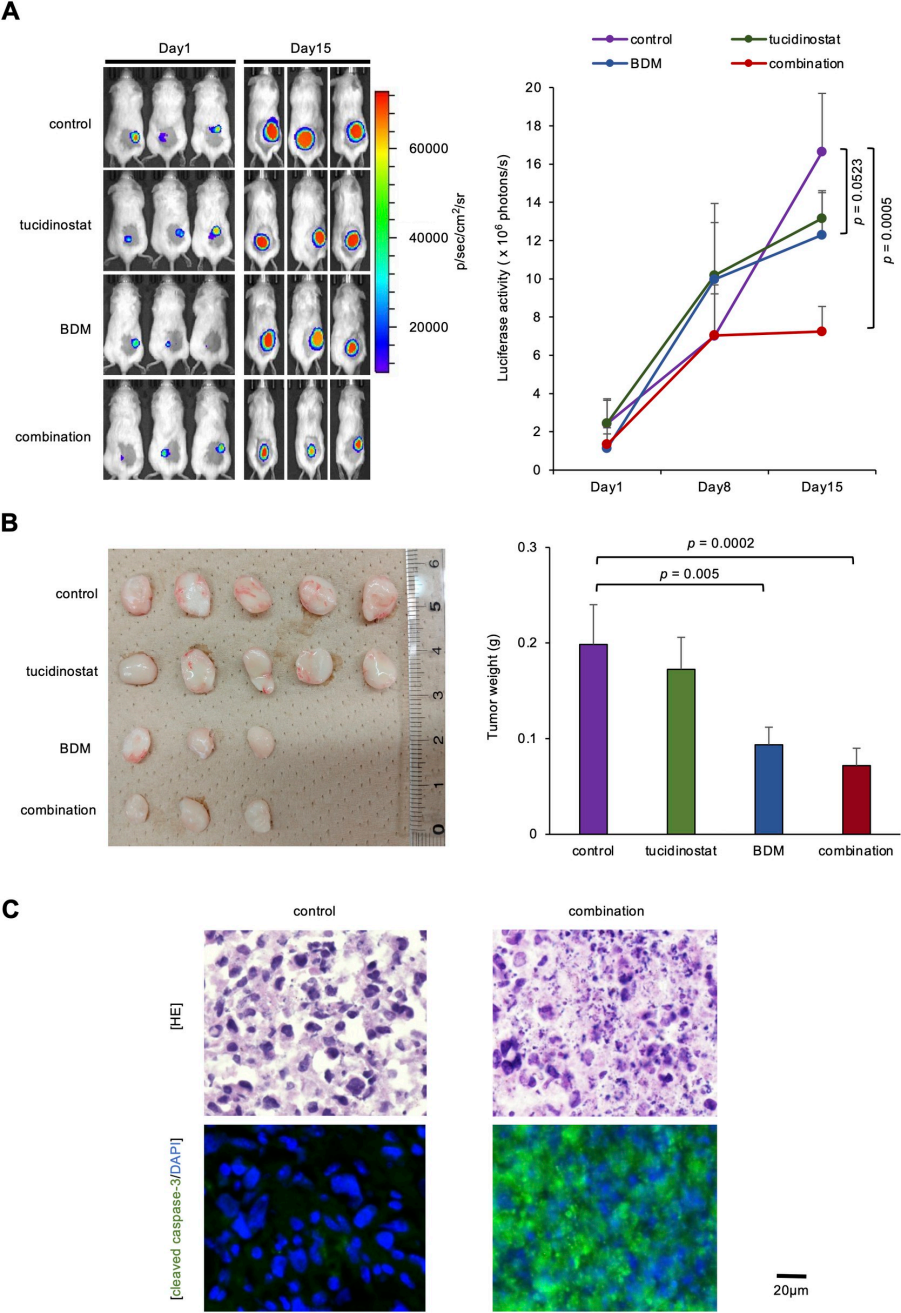
*In vivo* cytotoxicity toward ATL cells of bendamustine alone and in combination with tucidinostat. (A) We inoculated 5 × 10^5^ luciferase expressing MT2 cells subcutaneously into the right thigh of male NOD/SCID mice and randomized them into four treatment groups when measurable tumors developed over 5 x 10^5^ photons/s (day 1). Each group was treated with the vehicle alone (0.9% NaCl, orally, 5 times a week) (n = 5), tucidinostat alone (20 mg/kg, orally, 5 times a week) (n = 5), BDM alone (30 mg/kg, intraperitoneally, 3 times a week) (n = 3), or the combination of tucidinostat and BDM (n = 5 mice in each group) (n = 3) for 2 weeks. Left panel: tumor-derived luciferase activity was measured *ex vivo* by the IVIS Imaging System after D-luciferin injection. Representative photographs of NOD/SCID mice on day 1 and day 15 are shown (original magnification: × 2). Right panel: quantitative data of *in vivo* bioluminescence imaging shown in the left panel. The signal intensity is shown as photon units (photons/s). P values were determined by one-way analysis of variance (ANOVA) with a Student–Newman–Keuls multiple comparison test. (B) Left panel: representative photographs of tumors resected on day 30 (original magnification: × 2). Right panel: the means ± S.D. (bars) of the weights of resected tumors shown in the left panel. P values were determined by one-way analysis of variance (ANOVA) with a Student–Newman–Keuls multiple comparison test. (C) Upper panel: Tumor sections were prepared from mice and stained with hematoxylin eosin (HE). Lower panel: Serial tumor sections were immunostained for cleaved caspase-3 (green), then counterstained with 4’,6-diamidino-2-phenylindole (DAPI, blue). Only merged images are shown. Data shown are representative of multiple independent experiments.

To this end, we performed immunoblot analyses and revealed that the combination of BDM and tucidinostat increased caspase-3 activation with kinetics similar to the induction of cellular cytotoxicity (Fig 1D). This suggests that the increased caspase-induced apoptosis is an underlying mechanism of the combination of BDM and tucidinostat. Next, we validated the therapeutic effects of this drug combination *in vivo*. Luciferase-expressing MT2 cells were inoculated subcutaneously into the right thigh of nonobese diabetic/severe combined immunodeficient (NOD/SCID) mice. One week after transplantation, treatment was initiated with vehicle alone (0.9% NaCl) (n = 5), tucidinostat alone (n = 5), BDM alone (n = 3), or a combination (tucidinostat and BDM) (n = 3) was initiated in a randomly assigned group of mice. BDM alone and in combination with tucidinostat significantly slowed the growth of the tumors, as evidenced by luciferase activity tracked *ex vivo* (Fig 2A) and the size of the tumor excised 30 days after implantation (Fig 2B and S2A Fig). Tucidinostat alone was ineffective at the dose and in the schedule used. Histopathological examination of the resected tumors confirmed caspase-induced apoptosis by the combination of the two drugs (Fig 2C). We observed no significant changes in complete blood counts in mice but noted a moderate reduction in body weight in BDM-treated mice (S2B Fig). These results demonstrate the anti-tumor activity of BDM alone and in combination with tucidinostat *in vivo*. However, there is a limitation to this experiment. Since the MT2 cell line is an *in vitro* transformant of cord blood lymphocytes with leukemia cells from a ATL patient [18], further experiments using ATL cell lines are required to confirm the cytotoxicity of BDM toward ATL cells of BDM *in vivo*.

## Conclusions

We have shown cytotoxicity toward ATL cells of BDM alone and in combination with the novel agents, tucidinostat, valemetostat, and ABT-737. The combined *in vitro* effect of BDM and tucidinostat was reproduced in a murine model without any obvious hematological toxicity. Since the mid-2000s, development trials of novel agents for ATL have been active and some of these agents are already available in clinical practice. However, in a phase II trial in 23 patients with R/R progressive ATL, tucidinostat had an overall response rate of 30.4% and a median progression-free survival of only 1.7 months [25]. Our present results suggest that the combination of tucidinostat and BDM could additively prolong the survival of patients with R/R progressive ATL. The efficacy and safety of this combination are worthy of investigation in clinical settings.

## Supporting information

S1 Fig Dose-response curves and isobolograms for each cell line. (A) We cultured the indicated cell lines with various concentrations of bendamustine (BDM) for 72 h. Cell proliferation was estimated by the conversion of MTT (3-[4,5-dimethylthiazol-2-yl]-2,5- diphenyltetrazolium bromide) to formazan in viable mitochondria using a Cell Counting Kit (Wako Biochemicals, Osaka, Japan). In brief, cells were seeded in 96-well flat-bottomed microplates at a density of 1 × 104 cells per well and incubated at 37°C. After incubation, the absorbance of formazan was measured at a wavelength of 450 nm using a microplate reader (Bio-Rad Laboratories, Hercules, CA), and expressed as a percentage of the absorbance value of the corresponding control cells. The graph shows the means of triplicate samples; the S.D. was less than 10% and thus omitted. (B) S1T, TLSu, MT2, and ATN-1 cells were treated with BDM in combination with tucidinostat, valemetostat or ABT-737 in 96-well plates for 72 h. Dose-response curves of each combination were generated to construct nonconstant normalized isobolograms at IC_50_ using CompuSyn software (http://www.combosyn.com). FA and CI indicate fraction affected and combination index, respectively. The isobolograms shown are representative of at least three independent experiments. Combination indexes <0.8 and 0.8–1.2 indicate the synergism and additivity between the two drugs, respectively.
(TIF)

S2 Fig Tumor volume and evaluation of adverse events in mice. We inoculated 5 × 10^5^ luciferase expressing MT2 cells subcutaneously into the right thigh of male NOD/SCID mice and randomized them into four treatment groups when measurable tumors developed (day 1). Each group was treated with the vehicle alone (0.9% NaCl, orally, 5 times a week) (n-5), tucidinostat alone (20 mg/kg, orally, 5 times a week) (n = 5), BDM alone (30 mg/kg, intraperitoneally, 3 times a week)(n = 3), or the combination of tucidinostat and BDM (n = 3) for 2 weeks. (A) The means ± S.D. (bars) of the volumes (mm^3^) of resected tumors shown in the left panel of Fig 2. P values were determined by one-way analysis of variance (ANOVA) with a Student–Newman–Keuls multiple comparison test. (B) We measured the counts of white blood cells (WBC), hemoglobin, and platelets in the peripheral blood of recipient mice on day 30 of treatment. We measured body weights of mice on the indicated days. The means ± S.D. (bars) are shown (n = 3–5). P values were determined by Student’s t-test.
(TIF)

S1 File All relevant data within the manuscript. (DOCX)

S1 Raw images (TIF)
