# Supplementary figures and images for: Cytotoxicity of bendamustine, alone and in combination with novel agents, toward adult T-cell leukemia cells
Source: PLoS One. 2024 Sep 30;19(9):e0309533. doi: 10.1371/journal.pone.0309533 (PMC11441677; doi:10.1371/journal.pone.0309533)

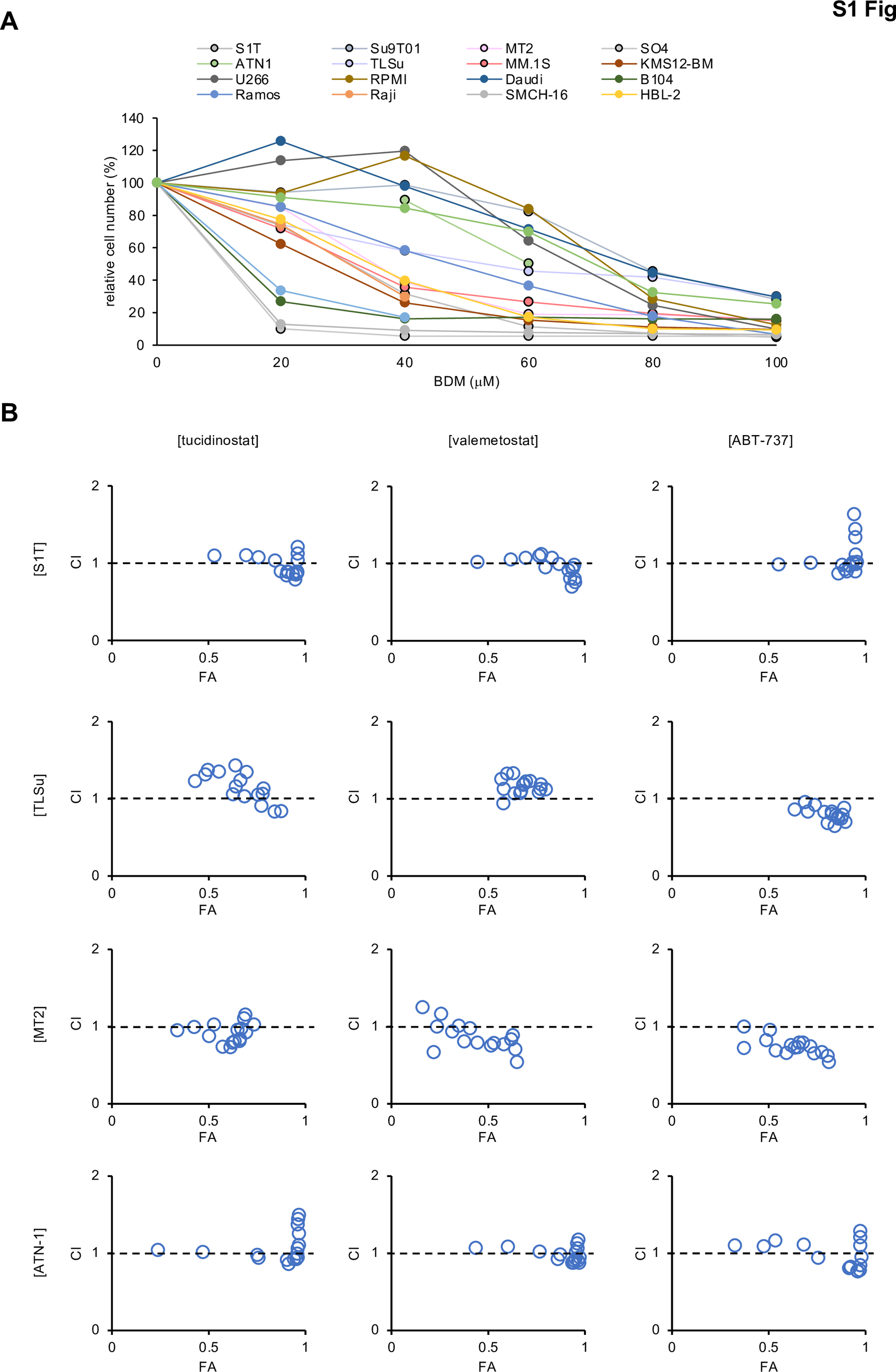

Supplement: S1 Fig — (A) We cultured the indicated cell lines with various concentrations of bendamustine (BDM) for 72 h. Cell proliferation was estimated by the conversion of MTT (3-[4,5-dimethylthiazol-2-yl]-2,5- diphenyltetrazolium bromide) to formazan in viable mitochondria using a Cell Counting Kit (Wako Biochemicals, Osaka, Japan). In brief, cells were seeded in 96-well flat-bottomed microplates at a density of 1 × 10^4^ cells per well and incubated at 37°C. After incubation, the absorbance of formazan was measured at a wavelength of 450 nm using a microplate reader (Bio-Rad Laboratories, Hercules, CA), and expressed as a percentage of the absorbance value of the corresponding control cells. The graph shows the means of triplicate samples; the S.D. was less than 10% and thus omitted. (B) S1T, TLSu, MT2, and ATN-1 cells were treated with BDM in combination with tucidinostat, valemetostat or ABT-737 in 96-well plates for 72 h. Dose-response curves of each combination were generated to construct nonconstant normalized isobolograms at IC50 using CompuSyn software (http://www.combosyn.com). FA and CI indicate fraction affected and combination index, respectively. The isobolograms shown are representative of at least three independent experiments. Combination indexes <0.8 and 0.8–1.2 indicate the synergism and additivity between the two drugs, respectively. (TIF) [file pone.0309533.s001.tif]

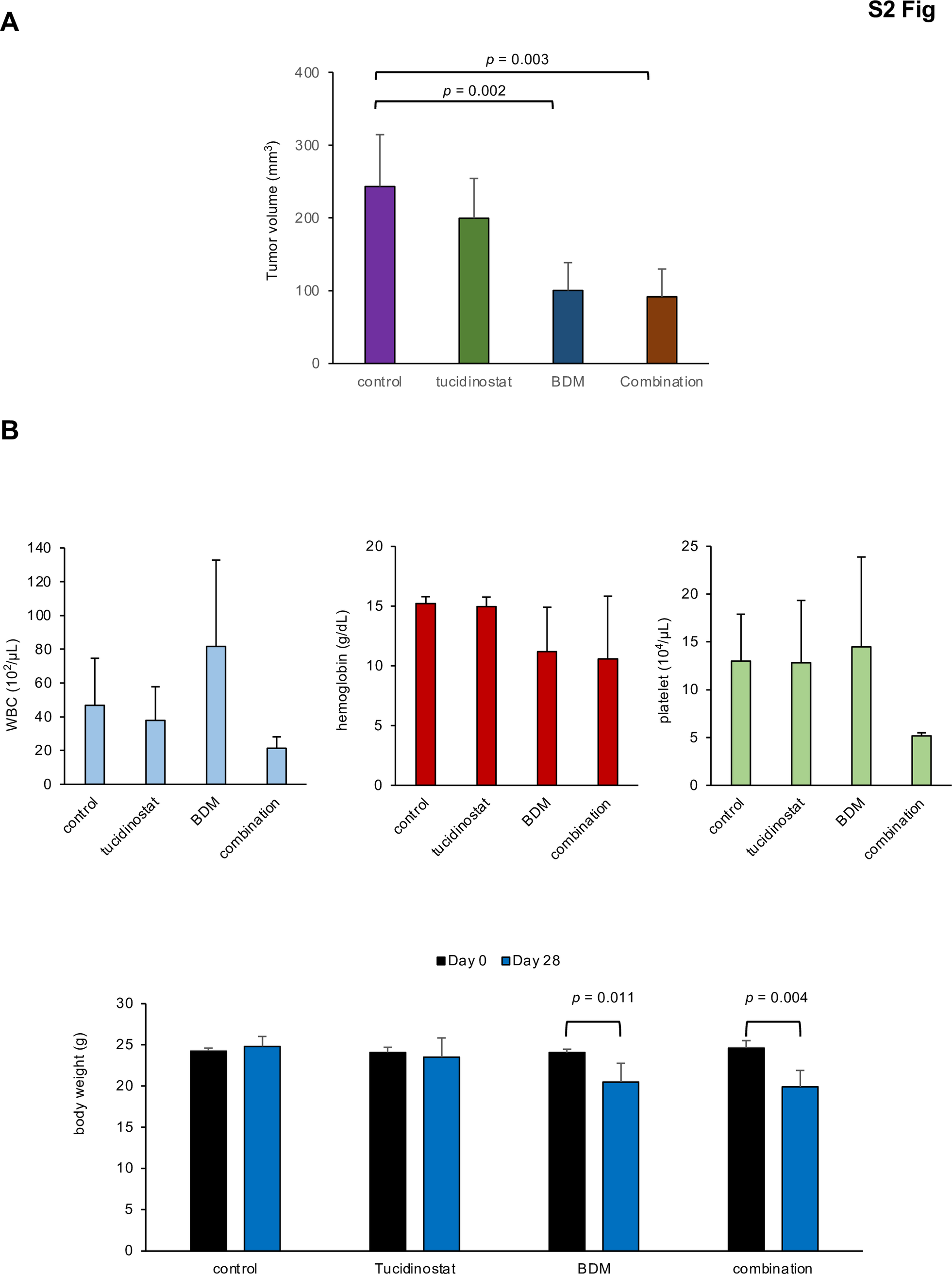

Supplement: S2 Fig — We inoculated 5 × 10^5^ luciferase expressing MT2 cells subcutaneously into the right thigh of male NOD/SCID mice and randomized them into four treatment groups when measurable tumors developed (day 1). Each group was treated with the vehicle alone (0.9% NaCl, orally, 5 times a week) (n-5), tucidinostat alone (20 mg/kg, orally, 5 times a week) (n = 5), BDM alone (30 mg/kg, intraperitoneally, 3 times a week)(n = 3), or the combination of tucidinostat and BDM (n = 3) for 2 weeks. (A) The means ± S.D. (bars) of the volumes (mm3) of resected tumors shown in the left panel of Fig 2. P values were determined by one-way analysis of variance (ANOVA) with a Student–Newman–Keuls multiple comparison test. (B) We measured the counts of white blood cells (WBC), hemoglobin, and platelets in the peripheral blood of recipient mice on day 30 of treatment. We measured body weights of mice on the indicated days. The means ± S.D. (bars) are shown (n = 3–5). P values were determined by Student’s t-test. (TIF) [file pone.0309533.s002.tif]

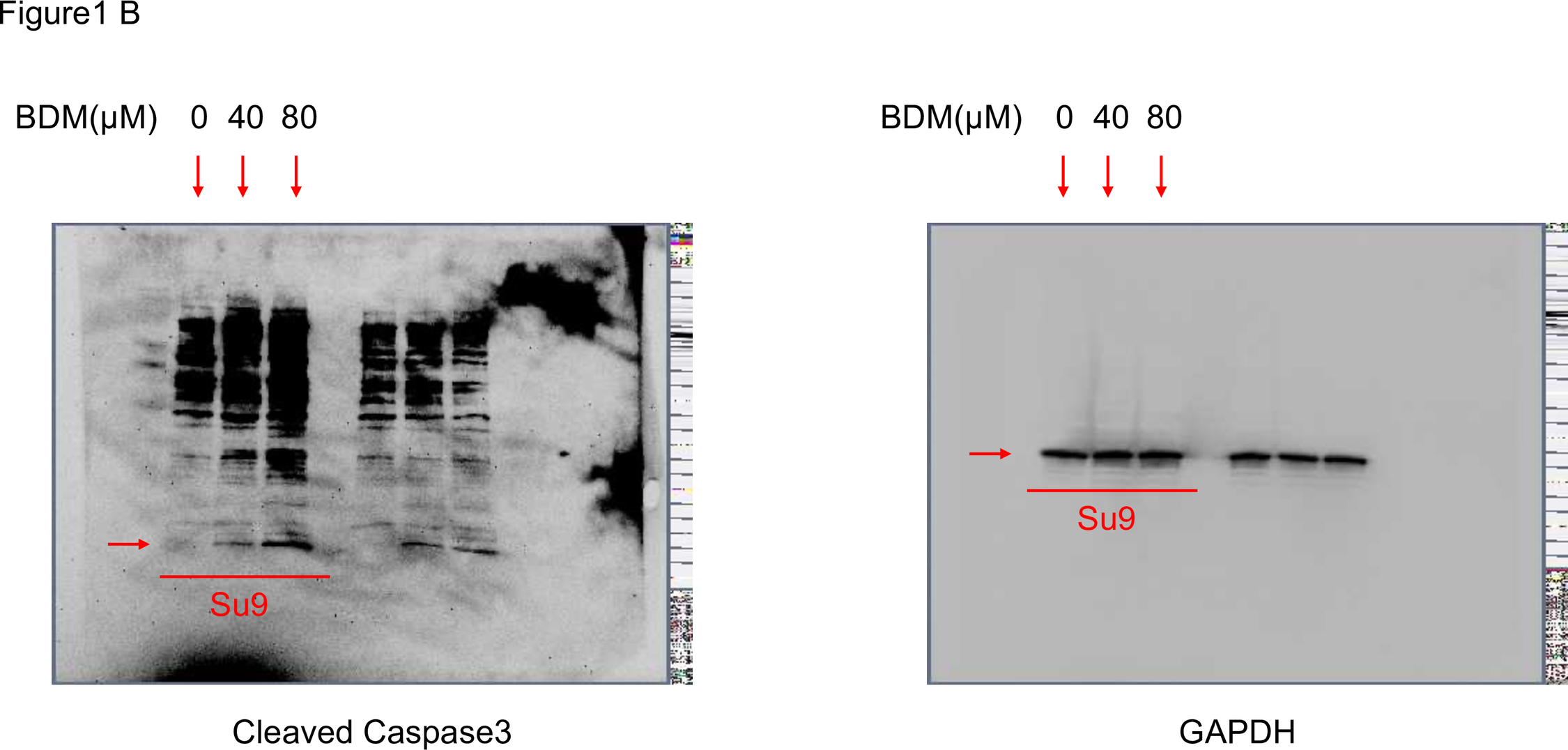

Supplement: S1 Raw images — (TIF) [file pone.0309533.s004.tif]
