# Supplementary material for: Cytotoxicity of bendamustine, alone and in combination with novel agents, toward adult T-cell leukemia cells
Source: PLoS One. 2024 Sep 30;19(9):e0309533. doi: 10.1371/journal.pone.0309533 (PMC11441677; doi:10.1371/journal.pone.0309533)
Supplement: S1 File — (DOCX) [file pone.0309533.s003.docx]

Minimal data set for Fig.1B

Minimal data set for Fig.1D

Minimal data set for Fig.2A

Minimal data set for Fig.2B

Minimal data set for S2 Fig.A

Minimal data set for S2 Fig.B :upper panel

Minimal data set for S2 Fig.B :lower panel
